# Nonlinear Absorption Properties of Cr_2_Ge_2_Te_6_ and Its Application as an Ultra-Fast Optical Modulator

**DOI:** 10.3390/nano9050789

**Published:** 2019-05-23

**Authors:** Peng-fei Ma, Wei Lin, Hua-nian Zhang, Shan-hui Xu, Zhong-min Yang

**Affiliations:** 1 State Key Laboratory of Luminescent Materials and Devices and Institute of Optical Communication Materials, South China University of Technology, Guangzhou 510640, China; shandapengfei@126.com (P.-f.M.); weilin_scut@163.com (W.L.); flxshy@scut.edu.cn (S.-h.X.); 2 Shandong Provincial Key Laboratory of Optics and Photonic Devices, School of Physics and Electronics, Shandong Normal University, Jinan 250014, China

**Keywords:** ferromagnetic insulator, ultra-fast optical modulation, fiber laser, femtosecond laser

## Abstract

In this manuscript, the nonlinear absorption properties of Cr_2_Ge_2_Te_6_ and its application in ultra-fast optical modulation are investigated. Typical parameters, namely, nonlinear absorption coefficient (β), saturation intensity, and modulation depth are measured to be ~1.66 × 10^−9^ m/W, 15.3 MW/cm^2^, and 5.8%, respectively. To investigate the feasibility of using the Cr_2_Ge_2_Te_6_ as an ultra-fast optical modulator, a ring-cavity passively mode-locked Er-doped fiber laser has been constructed. The output power/pulse, duration/pulse, and repetition rate/signal-to-noise ratios for the stable mode-locked operation are 2.88 mW/881 fs/19.33 MHz/48 dB, respectively, which proves that the Cr_2_Ge_2_Te_6_ has outstanding nonlinear optical properties and advantages in performing as an ultra-fast optical modulator. Further, the experimental results provide valuable references and open new avenues for developing two-dimensional, material-based, ultra-fast optical modulators and advanced photonic devices based on Cr_2_Ge_2_Te_6_.

## 1. Introduction

Over the past decade, layered two-dimensional (2D) materials have been used as a significant regime for exploiting potential optical functional devices, such as ultrafast photo-detectors [1], broadband optical modulators, and so forth [2,3,4,5]. In particular, benefitting from their unique optical properties, wideband optical modulators constructed by novel 2D-materials have significance in promoting the progress of ultra-fast lasers and their widespread related applications. As is known, graphene has opened the prelude of wide studies on 2D material-based ultra-fast fiber lasers [2,3,4,5,6]. Additionally, using MoS_2_ as a broadband modulator for generating an ultra-fast mode-locked laser was first demonstrated by Zhang et al. in 2014, which has inspired the investigations on various novel 2D saturable absorber materials due to their obvious advantages [6]. For example, compared with the conventional optical modulators fabricated via semiconductor saturable absorber mirrors (SESAMs), single walled carbon nanotubes (SWCNTs), or quantum dots [7,8], 2D materials have obvious advantages from the aspects of having wide absorption bands, easy and low-cost preparation, ultra-fast ps-level recovery time, high optical damage intensity, and low saturation intensity [4,5,6]. Different kinds of 2D materials, including topological insulators (TIs) [9,10,11,12], transition metal dichalcogenides (TMDs) [13,14,15,16,17,18,19], black phosphorus (BP) [20,21,22,23], and Mxenes [24,25], have been successively designed as optical modulators for generating ultrafast lasers. Nowadays, using 2D materials as modulators, mode-locked fiber lasers possess the properties of low cost, high efficiency, narrow pulse width, and compact structure. Further, the exploration of new 2D-materials with excellent nonlinear absorption properties is also required for extending the generation scope and diversity of ultra-fast lasers and advanced photonics.

Recently, as there are only a few rare ferromagnetic insulators, Cr_2_X_2_Te_6_ (X is silicon or germanium) has attracted particular attention [26,27,28,29,30,31,32,33,34]. Cr_2_X_2_Te_6_ not only has layered nearly-2D hexagonal structure, but also exhibits special transport magnetic, optical, and calculated electronic properties [27,28,29]. As a typical case, the Cr_2_Ge_2_Te_6_ ferromagnetic insulator has recently become the new focus [29,30,31,32,33,34]. As is reported, Cr_2_Ge_2_Te_6_ belongs to the space group of R3 and exhibits a relatively low bandgap value (0.7 eV) [30,31,32]. Previously, Cr_2_Ge_2_Te_6_ was successfully employed as a substrate for preparing TIs [33]. Gong et al. have reported that Cr_2_Ge_2_Te_6_ is an excellent Heisenberg ferromagnet, and was suitable for investigating fundamental spin behaviors and new photoelectric applications [34]. As is mentioned, compared with the mentioned 2D modulators, Cr_2_X_2_Te_6_ also exhibits a typical layered nearly-2D hexagonal structure and a suitable bandgap value (0.7 eV) [24]. Thus, Cr_2_Ge_2_Te_6_ is expected to have the same excellent nonlinear absorption properties and could be used as a wideband optical modulator for achieving ultra-fast lasers. To expand the ultra-fast optical applications of Cr_2_X_2_Ge_6_, investigations on the ultra-fast optical properties of Cr_2_X_2_Ge_6_ are of great significance. However, until now, the ultra-fast optical properties and modulation applications of the Cr_2_Ge_2_Te_6_ have not been investigated yet.

In our work, we investigate the nonlinear optical characteristics of the Cr_2_Ge_2_Te_6_ and employ it as an ultra-fast modulator based on an Er-doped fiber laser. By using Z-scan technique, obvious nonlinear absorption of the Cr_2_Ge_2_Te_6_ is observed and obtained. The saturation intensity and modulation depth are examined to be ~15.3 MW/cm^2^ and 5.8%, respectively. By using Cr_2_Ge_2_Te_6_ as the modulator, a stable passively mode-locked Er-doped fiber laser operating at 1561.57 nm is demonstrated. The narrowest pulse duration is measured to be ~881 fs. Our results reveal that Cr_2_Ge_2_Te_6_ has excellent saturable absorption characteristics and could be successfully used in ultra-fast fiber laser applications.

## 2. Fabrication and Characterization of the Cr_2_Ge_2_Te_6_-PVA Modulator

In our work, liquid stripping method and spin coating technology are employed to fabricate the Cr_2_Ge_2_Te_6_-polyvinyl alcohol (PVA) film-type optical modulator. The fabrication processes are listed below. In the first stage, 100 mg Cr_2_Ge_2_Te_6_ powder is added into 30 mL 30% alcohol and static soaking for 24 h. Then, in order to prepare the layered nanosheets, the mixture is set in a ultrasonic cleaner for 8 h. Then, the Cr_2_Ge_2_Te_6_-alcohol dispersion is centrifuged at a rate of 2000 rpm for 30 min to remove the precipitation. In the second stage, the 20 mL prepared Cr_2_Ge_2_Te_6_-alcohol dispersion is added into a 30 mL 5 wt.% PVA solution. The mixture is further ultrasonically-mixed for about 6 h. In the third stage, 80 μL Cr_2_Ge_2_Te_6_-PVA solution is transferred to the surface of a glass substrate through a spin-coated method and set into an oven for 24 h at 25 °C. In the final stage, a thin film is successfully fabricated for ultra-fast optical modulator application.

**Figure 1:**
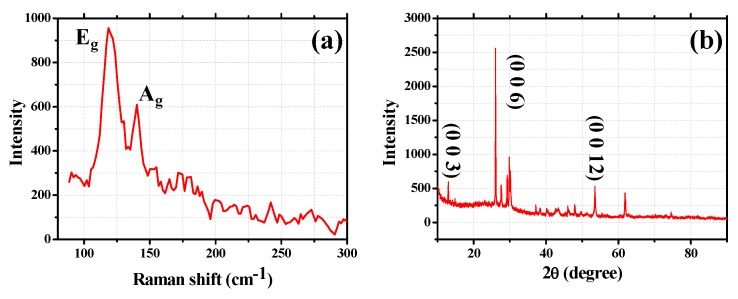
(**a**) The Raman spectrum of the Cr_2_Ge_2_Te_6_ nanosheets; (**b**) the X-ray diffraction of the Cr_2_Ge_2_Te_6_ nanosheets.

In the experiment, we have firstly measured the Raman characteristics of the Cr_2_Ge_2_Te_6_ powder. As described in Figure 1a, obvious Raman shifts of ~119 and ~140 cm^−1^, which, respectively, correspond to the typical A_g_ and E_g_ modes of the Cr_2_Ge_2_Te_6_, are observed [31]. The crystal structure of the Cr_2_Ge_2_Te_6_ powder is analyzed by using X-ray diffraction (XRD) and the diffraction XRD spectrum is depicted in Figure 1b. As provided in Figure 1b, typical peaks corresponding to the (003), (006), and (0012) planes in Cr_2_Ge_2_Te_6_ are recorded. The (003n) planes indicate that the layered Cr_2_Ge_2_Te_6_ powder with good crystallinity has been fabricated [30,31].

**Figure 2:**
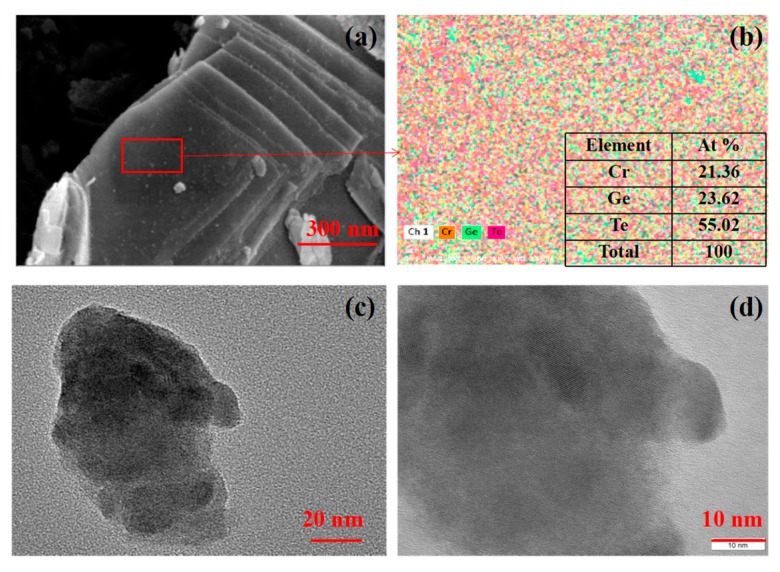
(**a**) The SEM image of the Cr_2_Ge_2_Te_6_ nanosheets; (**b**) the corresponding EDX image of the marked area of Figure 2a; (**c**) the TEM image of the Cr_2_Ge_2_Te_6_ nanosheets; (**d**) the HRTEM image of the Cr_2_Ge_2_Te_6_ nanosheets.

Further, the layered structure of Cr_2_Ge_2_Te_6_ is re-examined by employing a scanning electron microscope (SEM) (Sigma 500, Zeiss, Jena, Germany) and the measured result is shown in Figure 2a. From Figure 2a, it is shown that the Cr_2_Ge_2_Te_6_ fabricated in our experiment manifests obvious layered characteristics. Thus, multi- or single-layer nanosheets could be extracted based on the technique of ultrasonic stripping for optical modulator usage. Figure 2b shows the corresponding energy-dispersive X-ray spectroscopy (EDX) of the marked area in Figure 2a, and the colors associated with Cr, Ge, and Te are clearly expressed. The corresponding atomic ratio is nearly 2:2:6, which is compatible with the chemical formula of Cr_2_Ge_2_Te_6_. According to the measured results in Figure 2a,b, it could be indicated that relatively pure Cr_2_Ge_2_Te_6_ with a layered structure is achieved in the experiment. For further testing of the structure after the ultrasonic stripping, the transmission electron microscope (TEM) image of the Cr_2_Ge_2_Te_6_ nanosheet is detected by the TEM microscope (JEM-2100, Jeol, Tokyo, Japan) with a resolution of 20 nm. The typical result is shown in Figure 2c and an irregular layered structure is observed in the TEM image. Figure 2d presents the high resolution TEM (HRTEM) image of the sample captured with a scale of 10 nm. It is shown that the sample reveals a clear crystal lattice, indicating that layered Cr_2_Ge_2_Te_6_ with high crystallinity is obtained. In addition, as is shown, the morphology of nanosheets presented in Figure 2a dramatically differs from their morphology in Figure 2c,d. Figure 2a shows the SEM image of the powder Cr_2_Ge_2_Te_6_ sample. However, the liquid sample is prepared by soaking, ultrasonic oscillation, and centrifugation in turn to test TEM images shown in Figure 2c,d, and the preparation process has a great influence on the morphology of the nanosheets.

**Figure 3:**
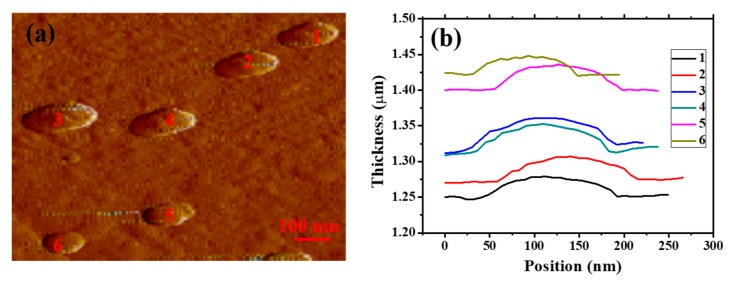
(**a**) The AFM image of the Cr_2_Ge_2_Te_6_ nanosheets; (**b**) the corresponding thicknesses of different samples.

The thickness of the Cr_2_Ge_2_Te_6_ material determines the saturation intensity and modulation depth, which will also influence its modulation properties in ultra-fast lasers. Consequently, the results of thickness characteristics of the Cr_2_Ge_2_Te_6_ are also examined by using an atomic force microscope (AFM, Bruker Multimode 8, Bruker, Karlsruhe, Germany). Six samples with relative individually uniform size and thickness are depicted. Figure 3b describes the thickness characteristics of the marked samples. The thicknesses of samples 2–5 are all approximately 23 nm and the thicknesses of samples 1 and 6 are about 21 nm. The overall results reveal that the layered samples with similar thicknesses are prepared, which ensures that the nonlinear optical characteristics of the Cr_2_Ge_2_Te_6_ modulator is controllable.

**Figure 4:**
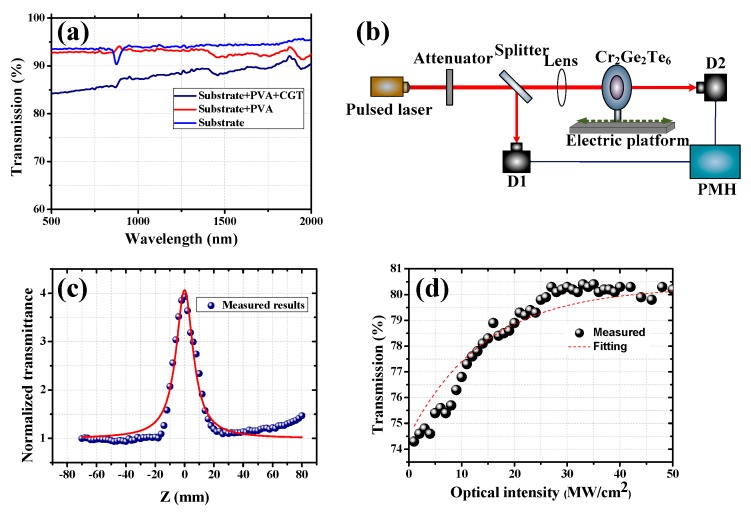
(**a**) Linear absorption spectra of the Cr_2_Ge_2_Te_6_-PVA modulator; (**b**) schematic of the Z-scan system; (**c**) open-aperture Z-scan results of the Cr_2_Ge_2_Te_6_-PVA film; (**d**) power-dependent nonlinear absorption property of the Cr_2_Ge_2_Te_6_-PVA film.

Figure 4a shows the optical transmission property of the Cr_2_Ge_2_Te_6_-PVA film, which is tested by a spectrophotometer (U-4100, Hitachi, Tokyo, Japan). For comparison, the transmission spectra of the substrate and bare PVA films on the substrate are also detected, as shown in Figure 4a. It is indicated that the PVA has little impact on the decrease of the transmittance, while the incorporation of Cr_2_Ge_2_Te_6_ (CGT) decreases the transmission from ~92% to ~88%. Consequently, the absorption is mainly induced by the Cr_2_Ge_2_Te_6_ material. Further, the nonlinear optical characteristic of the Cr_2_Ge_2_Te_6_-PVA film is examined by using an open-aperture Z-scan system (shown in Figure 4b). The 1064 nm picosecond pulsed laser with a pulse duration of ~25 ps under the pulse repetition rate of ~1 Hz is firstly attenuated by an attenuator and then injected into a 50:50 beam splitter. The reflection is captured by a power detector (D1) and the transmitted laser is focused by a lens with focal length of 150 cm, corresponding to a waist radius at the focal position ~49 μm. The Cr_2_Ge_2_Te_6_ sample is assembled on an electric platform and another power detector (D2) is employed to capture the transmitted beam. The captured results from D1 and D2 are displayed by using a double-channel power meter head (PMH). The laser intensity for the Z-scan setup can be tuned by controlling the input energy. In our work, we controlled the laser intensity level from MW/cm^2^ to GW/cm^2^. Figure 4c denotes the measured results obtained under the laser intensity of 13.8 GW/cm^2^ at the focal position and the fitting curve, which show that obvious nonlinear absorption properties could be achieved along with the change of beam intensity. Figure 4d shows the experimental and fitting results of the nonlinear absorption properties of the Cr_2_Ge_2_Te_6_-PVA film, which are tested by employing the widely-reported technique [13]. Further, based on the fitting curve of power-dependent nonlinear transmission, the saturation intensity and modulation depth can be achieved by the formula [13]:

(1) $$T(I)=1-T_{ns}-\Delta T\times \exp(-I/I_{sat})$$

where *T* is the transmission, *T_ns_* is the non-saturable absorbance, Δ*T* is the modulation depth, *I* is the input intensity of laser, and *I_sat_* is the saturation intensity. In our experiment, the non-saturable absorbance is 19.8%, and the saturation intensity and modulation depth are calculated to be 15.3 MW/cm^2^ and 5.8%, respectively. Additionally, it is noted that Cr_2_Ge_2_Te_6_ reduces the transmittance from 92% to 88%, i.e., by 4% (shown in Figure 4a). However, in Figure 4d, the unsaturated absorbance is indicated to be 19.8%; the difference is mainly due to the different pump source. The results shown in Figure 4a were tested by employing a continuous-wave laser as a pump source, meanwhile, a picosecond pulsed laser was used for testing the results shown in Figure 4d.

The following is the fitting equations for an open-aperture Z-scan curve [13]:

(2) $$T\left(z\right)=\left[1-\frac{\alpha_{0}LI_{s}}{I_{s}+I_{0}/\left(1+z^{2}/z_{0}^{2}\right)}\right]/\left(1-\alpha_{0}L\right)$$

where *z*, *z*_0_, *α*_0_*L*, *T*(*z*), *I*_0_, *I*_s_, are the sample positions relative to the focus position, the diffraction length of the beam, the modulation depth, the normalized transmittance at *z*, the peak on-axis intensity at focus, and the saturable intensity, respectively. In our experiment, the diffraction length of the beam, the peak on-axis intensity at focus, and the saturable intensity are 7.06 mm, 13.8 GW/cm^2^, and 15.3 MW/cm^2^, respectively. 

**Figure 5:**
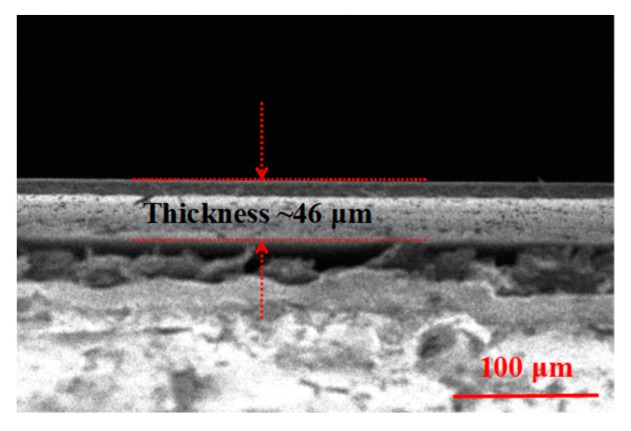
The SEM image of the Cr_2_Ge_2_Te_6_-PVA film.

In addition, based on the mentioned SEM (Sigma 500), the thickness of the Cr_2_Ge_2_Te_6_-PVA film (L) is tested to be about 46 μm (shown in Figure 5).

Based on the equations:

(3) $$I_{\mathrm{out}}=I_{\mathrm{in}}\times e^{-\alpha_{0}L}$$

(4) $$T_{0}=\frac{I_{\mathrm{out}}}{I_{\mathrm{in}}}=e^{-\alpha_{0}L}$$

where *I*_out_ and *I*_in_ are the output and input power used for testing the optical transmission property. Thus, combined with the data shown in Figure 4a (*T*_0_ = 83.37%), *α*_0_ is calculated to be 29.34 cm^−1^. Therefore, the value of the nonlinear absorption coefficient β is calculated to be about 1.66 × 10^−9^ m/W, which is in the same order of magnitude with TMDs. The results revealed above show that the Cr_2_Ge_2_Te_6_ exhibits suitable bandgap value sand excellent nonlinear absorption properties.

## 3. Experimental Setup

**Figure 6:**
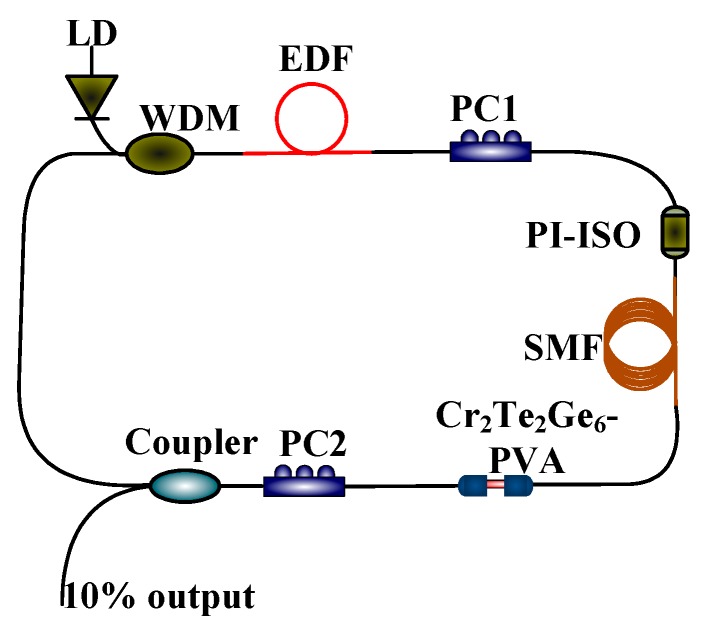
Experimental construction of the Cr_2_Ge_2_Te_6_-PVA-based Er-doped pulsed fiber laser.

In order to validate the feasibility of using the homemade Cr_2_Ge_2_Te_6_-PVA sample as an ultra-fast absorption modulator, an Er-doped and mode-locked laser is constructed based on an all fiberized ring cavity. The experimental construction is provided in Figure 6. A pigtailed laser diode (LD) with a central wavelength of 976 nm is employed to pump just ~36 cm highly Er-doped active fiber (EDF) (Liekki, Er-110, Nlight, Vancouver, WA, USA) with a core diameter of 4 μm via a wavelength division multiplexing (WDM) device. The absorption coefficient of the gain fiber is about ~60 dB/m at 976 nm and ~110 dB/m at 1530 nm. A polarization independent isolator (PI-ISO) guarantees the unidirectional transmission within the ring laser cavity. Two polarization controllers (PC1 and PC2) are incorporated into the laser cavity for polarization state adjustments. A 1 × 1 mm^2^ Cr_2_Ge_2_Te_6_-PVA film is cut off and transferred to the end of an optical connector for use as an ultra-fast absorption modulator. Finally, the output fiber laser is delivered from the 10% port of a 10:90 optical coupler (Coupler). In the experiment, a piece of single-mode fiber (SMF) is employed to adjust the dispersion characteristic, and the overall length of the passive fiber is about ~10.27 m. It is speculated that the final length of the ring fiber laser cavity is ~10.63 m, with a net dispersion value of ~0.205 ps^2^.

## 4. Results and Discussion

**Figure 7:**
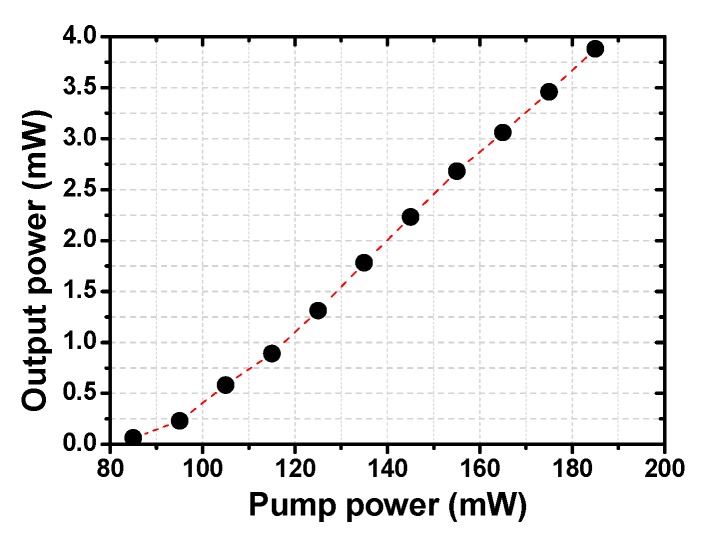
The relationship between the output power and the pump power of the continuous-wave operation.

Firstly, the Cr_2_Ge_2_Te_6_-PVA modulator is not inserted into the ring cavity. In this case, by adjusting the states of the PCs and tuning the pump power, the fiber laser is unable to operate in the stable self-Q-switched or mode-locked pulse state. The relationship of the continuous-wave output power and the pump power is shown in Figure 7. Under the pump power of 185 mW, the maximum output power and the slope efficiency are 3.88 mW and 3.86%.

**Figure 8:**
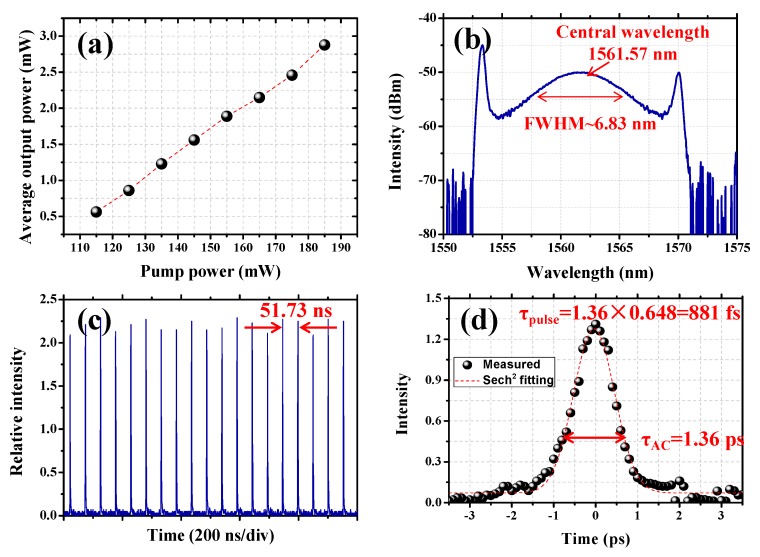
(**a**) Average output power scaling as a function of pump power; (**b**) optical spectrum at maximal output power; (**c**) typical pulse train of the output laser at maximal output power; (**d**) autocorrelation trace of the output laser at maximal output power.

Afterwards, the homemade modulator is added into the cavity for ultra-fast modulation. When the pump power is higher than 115 mW, a stable mode-locked pulse train is observed in the experiment, which indicates that the ultra-fast modulation effect is caused by the Cr_2_Ge_2_Te_6_-PVA. Figure 8a shows the average output power scaling characteristics along with the increase of pump power. A maximal output power of 2.88 mW could be achieved under the injected pump power of 185 mW, which corresponds to an optical conversion efficiency of ~1.56%. At maximal output power, the optical spectrum of the mode-locked ring fiber laser is shown in Figure 8b. It is shown that the soliton spectrum with typical Kelly side-band peaks is observed. The central wavelength is 1561.57 nm with a 3 dB spectral width of 6.83 nm. Figure 8c depicts one of the typical pulse trains at maximal output power. The pulse-to-pulse time is 51.73 ns and the pulse repetition rate is recorded to be ~19.33 MHz, exhibiting a cavity-length dependent property. The autocorrelation trace recorded by a autocorrelator (103XL) is shown in Figure 8d. By fitting with a sech^2^ function, the full width at half-maximum of the output pulse is calculated to be ~881 fs. The corresponding pulse energy and peak power are ~0.149 nJ and 169.1 W, respectively, due to the fact that the pulsed laser is delivered from the 10% port of a 10:90 optical coupler. Thus, the pulse energy and peak power in the modulator are estimated to be 1.34 nJ and 1.52 kW.

**Figure 9:**
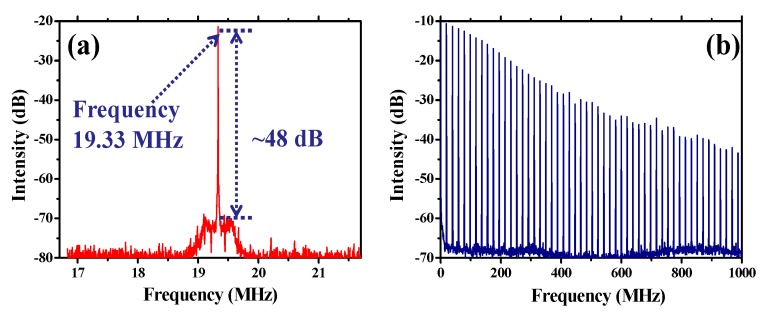
(**a**) The RF spectrum of the output laser at maximal output power recorded within a bandwidth of 5 MHz under a resolution of 1 kHz at the central frequency; (**b**) the RF spectrum within a spectral bandwidth of 1 GHz.

Figure 9a provides the radio frequency (RF) spectrum of the mode-locked operation at maximal pump power, which is recorded under a bandwidth of 5 MHz and a resolution of 1 kHz. It is obvious that the mode-locked operation is operating at the frequency of 19.33 MHz with a signal-to-noise ratio of ~48 dB. Figure 9b gives the RF spectrum recorded within a bandwidth of 1 GHz. The overall results provided in Figure 9a,b demonstrate that the mode-locked fiber laser operates in a stable state, which validates the capability of the homemade Cr_2_Ge_2_Te_6_-PVA saturable absorber as an ultra-fast optical modulator.

In Table 1, we provide typical comparative data of 2D material-based Er-doped mode-locked fiber lasers. As is shown, dates of typical 2D materials, including BP [20], TI [12] and TMDs [16,17,18,19] are provided. From the comparative data, we can draw the conclusion that Cr_2_Ge_2_Te_6_ exhibits relatively low saturable intensity and modulation depth, which lead to a small amount of energy within a fs-level pulse. All the obtained results and the comparative data prove Cr_2_Ge_2_Te_6_ has excellent nonlinear absorption properties and performance in acting as an ultra-fast modulator.

## 5. Conclusions

In conclusion, the Cr_2_Ge_2_Te_6_ sample with *β*, *I*_sat_, and *α_s_* values of 1.66 × 10^−9^ m/W, 15.3 MW/cm^2^, and 5.8%, respectively, was successfully prepared. By using the homemade Cr_2_Ge_2_Te_6_ sample as an ultra-fast optical modulator, the Er-doped ring fiber laser with stably mode-locked state was first demonstrated. A pulse duration of 881 fs under a repetition rate of 19.33 MHz was achieved. The experiment results show that Cr_2_Ge_2_Te_6_ has excellent nonlinear absorption properties, which could give a valuable reference for further investigating the applications of Cr_2_Ge_2_Te_6_ in ultra-fast optics.

## Figures and Tables

**Table 1:** Comparison of mode-locked Er-doped fiber lasers based on different 2D saturable absorbers (SAs).

SA	*α_s_*/*I_sat_* (MW·cm^−2^/%)	*λ_c_*/nm	*τ*	*E_pulse_*/nJ	Ref
BP	12/21	1559.5	670 fs	~6	[20]
Bi_2_Te_3_	28/6.2	1564.1	920 fs	15.36 pJ	[12]
In_2_Se_3_	7.8/4.5	1565	276 fs	2.03	[16]
MoTe_2_	9.6/25.5	1559	229 fs	2.14	[17]
WSe_2_	15.423/21.89	1557.4	163.5 fs	0.45	[18]
WS_2_	34.02/17.2	1561	246 fs	0.178	[19]
Cr_2_Ge_2_Te_6_	15.3/5.8	1561.59	881 fs	0.149	our

Note: *α_s_* = modulation depth; *I_sat_* = saturable intensity; *λ_c_* = Central Wavelength; *τ* = the pulse duration; *E_pulse_* = the pulse energy.
